# Hand hygiene monitoring technology: protocol for a systematic review

**DOI:** 10.1186/2046-4053-2-101

**Published:** 2013-11-12

**Authors:** Jocelyn A Srigley, David Lightfoot, Geoff Fernie, Michael Gardam, Matthew P Muller

**Affiliations:** 1 Institute of Health Policy, Management & Evaluation, University of Toronto, 155 College Street, Suite 425, Toronto, ON M5T 3M6, Canada; 2 Infection Prevention & Control, University Health Network, 585 University Avenue, Munk Building 9-800, Toronto, ON M5G 2C4, Canada; 3 Scotiabank Health Sciences Library, St. Michael’s Hospital, 30 Bond Street, Toronto, ON M5B 1W8, Canada; 4 Institute of Biomaterials and Biomedical Engineering, University of Toronto, 164 College Street, Room 407, Toronto, ON M5S 3G9, Canada; 5 Toronto Rehabilitation Institute, University Health Network, 550 University Avenue, Room 811, Toronto, ON M5G 2A2, Canada; 6 Department of Medicine, University of Toronto, Suite RFE 8-305, 200 Elizabeth Street, Toronto, ON, M5G 2C4, Canada; 7 Infection Prevention & Control, St. Michael’s Hospital, 4 Cardinal Carter North, Room 4-178, 30 Bond Street, Toronto, ON M5B 1W8, Canada

## Abstract

**Background:**

Healthcare worker hand hygiene is thought to be one of the most important strategies to prevent healthcare-associated infections, but compliance is generally poor. Hand hygiene improvement interventions must include audits of compliance (almost always with feedback), which are most often done by direct observation - a method that is expensive, subjective, and prone to bias. New technologies, including electronic and video hand hygiene monitoring systems, have the potential to provide continuous and objective monitoring of hand hygiene, regular feedback, and for some systems, real-time reminders. We propose a systematic review of the evidence supporting the effectiveness of these systems. The primary objective is to determine whether hand hygiene monitoring systems yield sustainable improvements in hand hygiene compliance when compared to usual care.

**Methods/Design:**

MEDLINE, EMBASE, CINAHL, and other relevant databases will be searched for randomized control studies and quasi-experimental studies evaluating a video or electronic hand hygiene monitoring system. A standard data collection form will be used to abstract relevant information from included studies. Bias will be assessed using the Cochrane Effective Practice and Organization of Care Group Risk of Bias Assessment Tool. Studies will be reviewed independently by two reviewers, with disputes resolved by a third reviewer. The primary outcome is directly observed hand hygiene compliance. Secondary outcomes include healthcare-associated infection incidence and improvements in hand hygiene compliance as measured by alternative metrics. Results will be qualitatively summarized with comparisons made between study quality, the measured outcome, and study-specific factors that may be expected to affect outcome (for example, study duration, frequency of feedback, use of real-time reminders). Meta-analysis will be performed if there is more than one study of similar systems with comparable outcome definitions.

**Discussion:**

Electronic and video monitoring systems have the potential to improve hand hygiene compliance and prevent healthcare-associated infection, but are expensive, difficult to install and maintain, and may not be accepted by all healthcare workers. This review will assess the current evidence of effectiveness of these systems before their widespread adoption.

**Study registration:**

PROSPERO registration number: CRD42013004519

## Background

Healthcare-associated infections (HAIs) lead to significant morbidity, mortality, and excess hospital costs. In 2002, it was estimated that HAIs affected 1.7 million patients in the US, resulted in 99,000 deaths, and were associated with excess healthcare costs of > USD 5 billion [1]. The morbidity, mortality, and costs associated with HAIs are increased further when they are caused by antibiotic-resistant organisms (AROs) [2].

In recent years, it has been increasingly realized that the majority of HAIs are preventable [3]. Healthcare worker (HCW) hand hygiene is considered to be one of the most important interventions for the prevention of HAIs and AROs [4,5]. However HCW hand hygiene is typically poor, with a median compliance of 40% [6], and few interventions have been demonstrated to result in significant and sustained improvement [4,5,7]. Currently, ‘multimodal’ hand hygiene programs are recommended, including education, improved access to alcohol-based hand sanitizer, workplace reminders, and regular and sustained audit and feedback of hand hygiene compliance [4,5,8]. Audit and feedback is essential as it is used both as a measure of hand hygiene compliance and as an effective improvement strategy [9]. However, audits are typically done by direct observation, which is labor-intensive, subjective, and ideally requires careful training of observers with regular measurement of inter-observer agreement to ensure reliability [4,10]. Furthermore, there is potentially a significant Hawthorne effect, whereby performance is higher during observation but returns to baseline as soon as observation stops. This is a particular problem given that, even with intensive monitoring, only a small fraction of hand hygiene opportunities are observed (typically <0.1%) [4,10].

Given these limitations, there is considerable interest in new technologies for audit and feedback of hand hygiene compliance, including electronic monitoring systems (EMS) and video monitoring systems (VMS). EMS vary widely, ranging from simple systems that track only the number of times that soap or hand sanitizer is dispensed to complex systems that integrate information on the timing of hand hygiene dispensing events and the location of the HCW in order to provide an estimate of hand hygiene compliance. VMS involve continuous video monitoring of the patient environment, with reviewers assessing and monitoring compliance. Both EMS and VMS can monitor hand hygiene on a continuous basis, allow measurement of compliance at the individual level, and may allow the provision of both group and individual feedback on a frequent basis. EMS reduces the subjectivity associated with the use of human auditors, while VMS may reduce this bias by having a smaller number of highly trained auditors external to the institution perform the monitoring. Some EMS also have the capability to provide real-time reminders to HCWs, such as auditory or vibratory signals to remind HCWs to perform hand hygiene if they enter or exit a patient room without activating the soap or sanitizer dispenser.

The most exciting potential benefit of these systems is that they may be an effective tool for improving hand hygiene compliance and reducing the incidence of AROs and HAIs, the ultimate goal of all hand hygiene promotion efforts. There are several mechanisms by which these systems can lead to improvement. The technology may create an enhanced Hawthorne effect as HCWs become aware that they are being monitored on a continuous basis. Additionally, the ability to provide more frequent and individual level feedback should improve compliance [11]. These systems are optimally designed to provide feedback and reminders on an ongoing basis and could contribute to achieving sustained improvements in hand hygiene compliance. Finally, some EMS systems provide real-time reminders to HCWs in a manner analogous to car alarms triggered when the seatbelt is not fastened. These reminders address the issue of ‘forgetting’, which has been identified as a common cause of missed hand hygiene opportunities [4].

Hospitals are under enormous pressure to improve hand hygiene and reduce HAIs. There are a number of choices in the area of hand hygiene monitoring and many systems are already being aggressively marketed. However, the systems vary widely in their design, few have been tested in rigorous controlled trials, and unanswered questions remain regarding their acceptability to HCWs and their real-world impact on hand hygiene and HAI rates [12]. There are currently only a small number of narrative reviews describe hand hygiene monitoring systems and the evidence supporting their efficacy [12,13]. As such, we propose a synthesis review to provide evidence as to whether currently tested systems can improve hand hygiene and/or reduce HAI rates and determine which types of systems are most effective.

### Research questions

#### Primary research question

1. Does hand hygiene monitoring technology, when compared to usual care and/or promotion efforts not involving monitoring technology, result in an increase in directly observed hand hygiene compliance?

#### Secondary research questions

2. Does hand hygiene monitoring technology, when compared to usual care and/or promotion efforts not involving monitoring technology, result in any of the following?

2.1 A reduction in ARO or HAI incidence

2.2 An increase in indirectly observed hand hygiene compliance as measured by volume of sanitizer dispensed or number of times dispenser activated

2.3 An increase in hand hygiene compliance as observed by the system itself

3. What is the mechanism by which successful monitoring technologies improve HCW hand hygiene? This will be determined through consideration of the following

3.1 Do systems that provide feedback lead to greater improvements in directly observed hand hygiene compliance and/or reductions in ARO/HAI than systems providing no feedback?

3.2 Do systems that provide real-time reminders lead to greater improvements in directly observed hand hygiene compliance and/or reductions in ARO/HAI than systems that provide delayed feedback?

3.3 Does the magnitude of improvement in hand hygiene compliance decrease over time, with ARO/HAI and directly observed compliance returning to baseline levels, suggesting an initial Hawthorne effect that wanes over time?

## Methods/Design

We prepared the systematic review protocol using guidance from the Preferred Reporting Items for Systematic Reviews and Meta-analyses Protocols (PRISMA-P) [14]. Our protocol has been registered in the PROSPERO database (CRD42013004519; available at: http://www.crd.york.ac.uk/PROSPERO/display_record.asp?ID=CRD42013004519#.UcroqDuyD_M).

### Eligibility criteria

The study designs included in this review will be randomized controlled trials (RCT), controlled and uncontrolled time series analyses, and other quasi-experimental designs including pretest/post-test studies. Studies will be included if the intervention described is either an EMS or VMS. Studies comparing EMS with VMS, or comparing different types of EMS/VMS, will only be included if they also include a comparison to a control group, which could include a parallel area not using EMS/VMS (for example, control arm of an RCT with multiple interventions) or baseline data obtained prior to implementing EMS/VMS (for example, baseline data from a time series analysis or pretest/post-test study). To qualify as an EMS, the described system must be able to perform at least one of the following functions: (1) count the number of hand hygiene events within a given geographic hospital area (for example, patient room, ward); (2) count the number of hand hygiene events associated with room entry and/or room exit events; (3) count the number of hand hygiene events associated with specific HCWs; or (4) estimate hand hygiene compliance using data from recorded hand hygiene events linked to HCW movement. To qualify as a VMS, the system must involve the recording of video within a patient care area with evaluation of the video by an internal or external observer using a specified definition of hand hygiene compliance. For both EMS and VMS, studies will be included regardless of whether they are used to provide feedback or real-time reminders to HCWs. However, studies will be excluded if the EMS or VMS is installed covertly, HCWs are not aware that they are being monitored, or the purpose of monitoring was exclusively to evaluate the impact of another intervention not related to the monitoring system and does not assess the impact of the monitoring system itself. Studies involving any HCW population will be included, whether in acute care or long-term care.

Studies must include at least one of the outcomes relevant to the objectives of the review. Thus, studies must include as an outcome either directly observed hand hygiene compliance, incidence of AROs or HAIs, or indirect estimates of hand hygiene compliance, which may be obtained from the monitoring system itself, or through estimates of volume of sanitizer or soap consumed, or count data measuring the number of hand hygiene events.

Studies using directly observed hand hygiene compliance as their outcome must use an accepted standard definition (for example, WHO [5], CDC [4], or similar). Studies reporting ARO or HAI incidence will be included if they report at least one HAI as an outcome, defined using standard CDC definitions or an accepted modification of CDC definitions (for example, catheter-associated urinary tract infection, hospital-acquired pneumonia, surgical site infection, catheter-associated bloodstream infection) or an ARO defined using standard microbiological definitions (for example, methicillin-resistant *Staphylococcus aureus* (MRSA), vancomycin-resistant enterococci (VRE)) [15].

There is a strong rationale for selecting directly observed hand hygiene compliance as the outcome for the primary research question, despite the fact that the ultimate goal of improvement in hand hygiene compliance is to reduce HAI and despite recognized limitations in the quality of directly observed hand hygiene compliance. First, our *a priori* knowledge of this field suggests that there will be little or no high quality evidence or studies linking EMS/VMS systems with reduction in HAIs and AROs in general and there is already substantive evidence linking improvements in hand hygiene compliance with reductions in HAIs/AROs, making this an important metric in its own right. Second, direct observation is currently considered the gold standard for the assessment of hand hygiene compliance. It allows hand hygiene performed by HCWs to be distinguished from hand hygiene performed by visitors or patients (as opposed to count or volume data) and is measured using standardized methodology across many studies, allowing comparisons between different EMS systems in terms of their impact on compliance. Compliance estimated by EMS systems would not be expected to be comparable between different systems as they typically detect different types of data and use different analytics and algorithms to provide an estimate of hand hygiene compliance.

Despite the above comments, we feel it is important to include studies using only a metric defined by the system itself to allow identification of all available systems that have the potential for improving hand hygiene. We do not want to exclude systems from consideration based on their failure to use direct observation, given that this measure is itself flawed and has been challenged. Thus systems that demonstrate a significant change in HCW behavior (even if not proven to improve directly observed hand hygiene) should be identified and flagged for further study using a more definitive outcome such as HAI incidence or directly observed hand hygiene. These studies would be considered separately, however, and in a qualitative fashion.

Studies included in the analyses to determine the mechanisms by which monitoring technologies lead to change must meet the inclusion criteria as described above. A subset of these studies that report on outcomes over time (hand hygiene compliance and/or HAIs/AROs) will be used to determine whether there may be a Hawthorne effect leading to change. Studies of systems that use real-time reminders, delayed feedback, or no reminders/feedback will be compared to determine if improvements in compliance or ARO/HAI incidence are related primarily to monitoring or result primarily from feedback and/or reminders.

Only published, peer-reviewed studies will be included. Inclusion will not be limited by language. Studies will not be excluded based on poor methodology as long as they meet the minimum requirements for study design described above; study quality will be assessed and taken into account as described below.

The draft eligibility criteria can be found in Appendix 1.

### Information sources

Database searches will be performed in MEDLINE, EMBASE, CINAHL, Web of Science, and the Cochrane Central Register of Controlled Trials (CENTRAL). All databases will be searched from inception until the present. We will conduct a hand search of a set of infection control journals most likely to publish articles on this topic including: *Infection Control and Hospital Epidemiology*, *Journal of Hospital Infection*, and *The American Journal of Infection Control*. A search of the proceedings of the Society of Healthcare Epidemiology of America will be performed. The reference lists of included studies and relevant review articles will also be searched. Finally, Google will be searched to identify unpublished and grey literature that may suggest publication bias.

### Literature search

An experienced information specialist (DL) will conduct comprehensive literature searches. Search strategies will be developed using medical subject headings (MeSH) and text words related to hand hygiene, compliance/monitoring, and electronic systems. The draft search strategy for MEDLINE can be found in Appendix 2.

### Study selection process

The titles and abstracts of all retrieved studies will be independently reviewed by two reviewers (JAS and MPM) based on the eligibility criteria described above. The complete article will then be obtained for any study deemed to potentially meet the inclusion criteria by either reviewer. All complete articles will again be reviewed independently by two reviewers (JAS and MPM) and their eligibility determined based on the criteria described above. If a difference of opinion occurs between the two reviewers that cannot be resolved, a third reviewer (MG) will review the article and make a final determination regarding inclusion. The rationale for exclusion will be documented for all excluded articles.

### Data items

The data abstracted will include the study setting (type of facility, number of rooms or wards, type of wards), study participants (number, professions of HCWs, age, years of experience), study design (RCT or time series or quasi-experimental design; control group, if any; use of cross-over, switching replications, stepped wedge or other quasi-experiment design elements intended to reduce bias; installation period, enrolment period, data collection period), study intervention (type of EMS or VMS, type and frequency of feedback, type and frequency of real-time reminders) and co-interventions (education or training provided, other simultaneous interventions or promotions in addition to the EMS/VMS), study-specific outcomes (for example, directly or indirectly observed hand hygiene compliance, HAIs, AROs, acceptability of technology to staff, use of direct observation, training of direct observers, measurement of inter-observer agreement, definition of hand hygiene compliance, definition and type of HAI, definition and type of ARO).

### Data collection process

A data collection form will be drafted, piloted, and modified as necessary. Two reviewers (JAS and MPM) will complete the form independently for each included study. If there are discrepancies that cannot be resolved by discussion, a third reviewer (MG) will decide. Data will be collected using Microsoft Excel.

### Risk of bias appraisal

Study quality and risk of bias will be assessed independently by two investigators (JAS and MPM), with disputes resolved by a third reviewer (MG). All identified RCTs, time series analyses, and controlled pretest/post-test studies will be assessed using the Cochrane Effective Practice and Organization of Care Group Risk of Bias Assessment Tool [16]. Because we anticipate that the majority of studies will be uncontrolled pretest/post-test studies, we will also use guidelines developed specifically for the evaluation of these study designs [17,18].

There are also specific biases that can be anticipated in studies of EMS/VMS. In particular, there is the potential for co-interventions to occur when EMS/VMS are implemented. EMS implementation requires substantial initial training and education of HCWs as well as ongoing training and assistance, which could alter hand hygiene behavior independent of the EMS itself. Ideally, a similar intensity of hand hygiene education should be provided on a control unit. Implementation of EMS systems may also include elements such as portable hand sanitizer dispensers that could affect HCW behavior independent of the effect of monitoring and feedback. Again, in the ideal study these elements would also be provided to HCWs in a control group. We will carefully assess for these potential sources of bias and, where the nature and extent of education and training is uncertain, may contact the study authors for clarification.

In addition to the specific biases that may be present within individual studies, there is a significant risk for publication bias in this area, as data supporting the use of novel technologies are likely of greater interest for publication than negative studies. Additionally, as many systems are developed by industry, financial incentives likely favor the submission of positive rather than negative studies. We will attempt to minimize the risk by including a broad search, including non-English publications, and by searching the grey literature and conference proceedings for unpublished studies. If sufficient, similar studies are available to allow meta-analysis, a funnel plot will be assessed to look for publication bias. We will also assess trial registries to determine whether there are any missing studies, suggesting potential publication bias, and will compare the methods of published studies with their original protocols to assess for outcome reporting bias.

### Synthesis of results

The synthesis of results will begin with a descriptive summary of included studies, focusing on study methodology, interventions, participants, and outcomes, as well as a summary table on risk of bias in the included studies.

The effectiveness of the monitoring systems in the included studies will then be summarized as the mean change in outcome (%). The comparative effectiveness results will be presented according to the research questions, with each of the outcomes reported separately (directly observed hand hygiene compliance, HAI/ARO rates, indirect measures of hand hygiene compliance, and hand hygiene compliance as monitored by the system itself).

For this systematic review, we do not anticipate the performance of a formal meta-analysis as we expect that there will be significant heterogeneity in terms of study designs, types of technology implemented, the frequency and nature of feedback, and the types and definitions of outcomes utilized, which would preclude the calculation of summary measures. However if we do identify at least two comparable studies that describe a similar type of monitoring technology and report on the same outcomes, we will calculate summary measures based on the percent change in hand hygiene compliance and/or HAI/ARO rates. The I^2^ statistic will be used to assess heterogeneity, and meta-analysis will not be performed if I^2^ is >50%. The possible reasons for heterogeneity will be explored, including study design, HCW population, clinical setting, and features of the technology. If meta-analysis can be performed, the mean difference will be used as the summary statistic since the outcome data are continuous. If there is moderate heterogeneity (I^2^ of 30% to 50%), a random effects model will be used for meta-analysis [19].

The next step will be a narrative synthesis based on the Economic and Social Research Council (ESRC) guidance report [20]. This will include proposing a theoretical model of how hand hygiene monitoring technology may improve hand hygiene compliance and in what circumstances, describing patterns of effect size and direction in included studies, exploring factors that might explain differences across studies, and assessing the strength of the evidence. We plan *a priori* to explore the two possible mechanisms by which EMS/VMS may result in improved hand hygiene compliance, namely an enhanced Hawthorne effect due to the continuous monitoring and the provision of feedback and/or reminders. We will assess whether there is any apparent relationship between the degree of improvement in compliance identified and the duration of data collection to determine whether the initial benefits of EMS/VMS decline over time as HCWs become accustomed to ongoing monitoring and thus the impact of the Hawthorne effect wanes. We will also determine whether there is any relationship between the reported efficacy of each system and their use of feedback (that is, studies without feedback *vs*. studies with feedback *vs.* studies providing real-time reminders). Finally, we will also evaluate study quality in relation to the demonstrated efficacy of each system or type of system for each of the primary and secondary outcomes.

We will evaluate the overall quality of evidence using the GRADE approach [21] if the synthesized data allows - that is, if an effect estimate for any given specific outcome of interest is reported by two or more controlled studies per each between-intervention comparison.

## Discussion

This systematic review will assess whether hand hygiene monitoring technology improves hand hygiene compliance and reduces HAI rates. Given the importance of hand hygiene in HAI prevention, an intervention capable of improving and sustaining hand hygiene performance has an enormous potential to save lives, reduce morbidity and length of stay, and minimize healthcare costs. Conversely, the misdirection of infection control resources and budgets towards unproven and ineffective technology; or the inadvertent selection of a specific technological product that lacks one or more key element and may therefore lack efficacy, has the potential to worsen, rather than improve, this situation.

In this context, we believe this systematic review is ‘post-mature’ and is urgently needed as hospitals in the US and Canada are already beginning to adopt hand hygiene monitoring technology despite a lack of systematic evidence of its benefit. Our review has the potential to accelerate the momentum behind the uptake of this novel form of technology or to slow it, depending on the nature of the results; it may also help direct consumers (that is, hospitals, health systems, governments) towards those products that are most likely to be effective. If we identify one or more monitoring systems clearly associated with sustained reductions in hand hygiene compliance and/or HAIs, our review has the potential not only to change practice on a broad scale but to dramatically improve patient safety and outcomes.

## Appendix 1: Draft eligibility criteria

1. Does this study include an intervention that is either an electronic monitoring system (EMS) or video monitoring system (VMS)?

YES, EMS if the system meets at least one of the following criteria: _____

1. Counts the number of hand hygiene events within a given geographic hospital area _____

2. Counts the number of hand hygiene events associated with room entry and/or room exit events _____

3. Counts the number of hand hygiene events associated with specific HCWs _____

4. Estimates hand hygiene compliance using data from recorded hand hygiene events linked to HCWs movement _____

YES, VMS if the system involves recording of video with evaluation of the video by an observer using a specified definition of hand hygiene compliance _____

NO _____

UNCLEAR _____

2. Was the EMS or VMS installed overtly, were HCWs aware that they were being monitored, and/or was impact of the monitoring system assessed?

YES _____

NO _____

UNCLEAR _____

3. Is the hand hygiene technology being compared to usual care and/or promotion efforts not involving monitoring technology?

YES _____

NO _____

UNCLEAR _____

4. Is the study a randomized controlled trial or quasi-experimental design?

YES _____

NO _____

UNCLEAR _____

5. Does this study report any of the relevant outcomes (hand hygiene compliance as measured by direct observation or by other metrics, HAI, and/or ARO incidence)?

YES _____

NO _____

UNCLEAR _____

If the answer to any of these questions is NO based on titles and abstracts, the study will be excluded. After the first stage of screening, the full text of potentially eligible studies will be obtained for further review. If the answer to any of these questions is NO based on full text, the study will be excluded. All other studies will be included. We will keep track of citations that have potentially relevant material (for example, review articles) and will scan their reference lists to ensure all studies have been captured.

## Appendix 2: Draft Medline search strategy

Database: Ovid MEDLINE(R) In-Process & Other Non-Indexed Citations and Ovid MEDLINE(R) <1946 to Present>

--------------------------------------------------------------------------------

1 exp Iatrogenic Disease/ (12646)

2 exp Cross Infection/ (45840)

3 nosocomial.mp. (21210)

4 iatrogenic$.mp. (27129)

5 exp Vancomycin Resistance/ (2869)

6 VRE.mp. (1865)

7 exp Methicillin-Resistant Staphylococcus aureus/ (6427)

8 mrsa.mp. (13766)

9 exp hand/ (66640)

10 hand.mp. (297499)

11 or/1-10 (414583)

12 exp infection control/ (49081)

13 pc.fs. [prevention and control as a floating subject heading] (998131)

14 exp anti infective agents/ (1244944)

15 exp Decontamination/ (3345)

16 disinfect$.mp. (30342)

17 or/12-16 (2181031)

18 12 and 17 (76024)

19 exp hand hygiene/ [includes the MeSH Hand Disinfection] (4492)

20 (hand adj2 wash$).mp. [mp = title, abstract, original title, name of substance word, subject heading word, keyword heading word, protocol supplementary concept, rare disease supplementary concept, unique identifier] (1782)

21 (hand adj2 hygiene$).mp. [mp = title, abstract, original title, name of substance word, subject heading word, keyword heading word, protocol supplementary concept, rare disease supplementary concept, unique identifier] (1971)

22 (hand adj2 clean$).mp. [mp = title, abstract, original title, name of substance word, subject heading word, keyword heading word, protocol supplementary concept, rare disease supplementary concept, unique identifier] (218)

23 (hand adj2 sanitiz$).mp. [mp = title, abstract, original title, name of substance word, subject heading word, keyword heading word, protocol supplementary concept, rare disease supplementary concept, unique identifier] (162)

24 (hand adj2 disinfect$).mp. [mp = title, abstract, original title, name of substance word, subject heading word, keyword heading word, protocol supplementary concept, rare disease supplementary concept, unique identifier] (4688)

25 or/19-24 (6625)

26 18 or 25 (76904)

27 exp population surveillance/ (50191)

28 exp population surveillance/ (50191)

29 surveillance.mp. (136275)

30 monitor$.mp. (606051)

31 feedback.mp. (95605)

32 alarm.mp. (6107)

33 or/27-32 (819570)

34 26 and 33 (7684)

35 exp automation/ [includes MeSH robotics] (25847)

36 automated system.mp. (2316)

37 automatic$.mp. (76950)

38 sensor$.mp. (242568)

39 RFID.mp. (423)

40 exp Radio Frequency Identification Device/ (220)

41 exp Electronics/ (25648)

42 exp Video-Audio Media/ (5502)

43 (monitor adj2 computer$).mp. [mp = title, abstract, original title, name of substance word, subject heading word, keyword heading word, protocol supplementary concept, rare disease supplementary concept, unique identifier] (656)

44 (monitor adj2 video$).mp. [mp = title, abstract, original title, name of substance word, subject heading word, keyword heading word, protocol supplementary concept, rare disease supplementary concept, unique identifier] (481)

45 (monitor adj2 electr$).mp. (812)

46 (system$ adj2 computer$).mp. (38430)

47 (system$ adj2 video$).mp. (2522)

48 (system$ adj2 electr$).mp. (16530)

49 exp tape recording/ [includes Videotape Recording] (14287)

50 computer.ti,ab. [title or abstract] (158475)

51 video.ti,ab. [title or abstract] (47539)

52 exp computer systems/ (136153)

53 or/35-48 (409583)

54 34 and 53 (268)

55 remove duplicates from 55 (252)

## Competing interests

The authors report no financial or non-financial conflicts of interest.

## Authors’ contributions

JAS and MPM contributed to the conception and design of the review. JAS wrote the draft protocol and registered the protocol with the PROSPERO database. JAS, DL, MG, and MPM will be involved in data acquisition. JAS, GF, MG, and MPM will synthesize and interpret results. All authors were involved in the editing of this protocol and have given their approval for publication.
